# Brain responses to body image stimuli but not food are altered in women with bulimia nervosa

**DOI:** 10.1186/1471-244X-13-302

**Published:** 2013-11-15

**Authors:** Frederique Van den Eynde, Vincent Giampietro, Andrew Simmons, Rudolf Uher, Chris M Andrew, Philippe-Olivier Harvey, Iain C Campbell, Ulrike Schmidt

**Affiliations:** 1 Department of Psychological Medicine, Section of Eating Disorders Institute of Psychiatry, King’s College London, London, UK; 2 Eating Disorders Program, Douglas Mental Health University Institute and McGill University, Montréal, Québec, Canada; 3 Institute of Psychiatry, Department of Neuroimaging, King’s College London, London, UK; 4 NIHR Biomedical Research Centre for Mental Health at South London and Maudsley NHS Foundation Trust and Institute of Psychiatry, King’s College London, London, UK; 5 Department of Psychiatry, Dalhousie University, Halifax, NS, Canada; 6 Douglas Mental Health University Institute and McGill University, Montréal, Québec, Canada; 7 Institute of Psychiatry, King’s College London, PO Box 59, De Crespigny Park, SE5 8AF, London, UK

**Keywords:** Functional magnetic resonance imaging, Bulimia nervosa, Insula, Anxiety, Craving

## Abstract

**Background:**

Research into the neural correlates of bulimia nervosa (BN) psychopathology remains limited.

**Methods:**

In this functional magnetic resonance imaging study, 21 BN patients and 23 healthy controls (HCs) completed two paradigms: 1) processing of visual food stimuli and 2) comparing their own appearance with that of slim women. Participants also rated food craving and anxiety levels.

**Results:**

Brain activation patterns in response to food cues did not differ between women with and without BN. However, when evaluating themselves against images of slim women, BN patients engaged the insula more and the fusiform gyrus less, compared to HCs, suggesting increased self-focus among women with BN whilst comparing themselves to a ‘slim ideal’. In these BN patients, exposure to food and body image stimuli increased self-reported levels of anxiety, but not craving.

**Conclusions:**

Our findings suggest that women with BN differ from HCs in the way they process body image, but not in the way they process food stimuli.

## Background

People with bulimia nervosa (BN) engage in binge-eating (i.e. over-eating with a feeling of losing control) and compensatory behaviors such as self-induced vomiting, the use of laxatives or diuretics or excessive exercising. It affects approximately 1% of young women [1] and treatment outcomes such as remission rates are modest [2,3]. The neurobiological underpinnings of BN are poorly understood; Functional magnetic resonance imaging (fMRI) studies focused on self-regulatory [4,5] and reward processes [6-8] have identified differences in fronto-striatal functioning in people with BN and these may underlie core BN symptomatology such as binge eating, purging and undue body weight and shape preoccupation and dissatisfaction. However, fMRI research in BN is still at an early stage [9]; for example, replication of findings is rare and knowledge on the processing of visual food and body image cues is based on different patient cohorts.

Processing of food images in people with BN has been examined in 5 fMRI studies (n_range_ = 8–20) by comparing responses to food and non-food images [10-14]. However, there is a lack of consistency in the findings. Uher et al. (2004) reported that, compared to healthy controls (HCs), BN patients showed increased left medial prefrontal cortex activation, combined with a decreased left (dorso)lateral prefrontal cortex (DLPFC) activation in response to visual food cues [10]. Joos et al. (2011) also found lower activation levels in people with BN in a larger region comprising parts of the anterior cingulate cortex (ACC) and lateral prefrontal cortex [12]. Weak activation of the DLPFC activation in response to food stimuli has been interpreted as poor ‘top-down’ self-regulatory control in people with BN [10,12]. A number of findings have not yet been replicated, e.g. reduced activation in the right middle temporal lobe and mid-cingulate cortex [12], reduced left visual cortex activation [15], and reduced inferior parietal lobe and post-central gyrus activations [14]. Other findings appear to be in ‘opposite directions’, e.g. Brooks et al. (2011) reported decreased bilateral insula activation in people with BN, while Schienle et al. (2009) reported increased right insula activation [11]. Furthermore, the increased bilateral ACC activation in BN seen by Schienle et al. (2009) contrasts with the finding by Joos et al. (2011) in this region. Thus, there is insufficient evidence to state that fronto-striatal circuit dysfunctions are associated with food/binge-eating related BN psychopathology. Part of the problem may relate to the fact that all previous studies used a non-food (e.g. stationary) control condition rather than a contrast of the food stimuli with a ‘low level baseline’ (LLB) condition, e.g. looking at a cross, which has been used in HC studies [16]. It is possible that women with BN already differentially respond to certain control conditions, i.e. non-food [or non-body image [17]], the contrast between the food condition and a LLB may clarify the differential involvement of fronto-striatal areas in food processing between women with and without BN.

Undue body image concerns are another major pathological feature of BN and hence brain activation patterns associated with the processing of visual presentations of one’s own and other women’s bodies have been studied using various fMRI paradigms. One repeated finding is that women with BN engage the medial prefrontal cortex less than HCs when presented with body stimuli [18-21]. However, differences in brain activation patterns between women with and without BN have also been seen in several regions (temporal, parietal and occipital) [18-22]. Methodological variations such as the use of different stimuli (e.g. own or others’ bodies) and different verbal instructions associated with these visual stimuli [20] may have contributed to these divergent findings. Responses to stimuli involving evaluation of one’s own or comparative evaluation of another body involve both interoceptive awareness and emotional elements. The insula, which is involved in interoceptive awareness [23,24], has been implicated in the pathophysiology of eating disorders [25,26]. In people with BN, the insula has been reported to be involved in the processing of thinner own-body representations [20]. Evaluation of own physical appearance in comparison to ‘slim ideals’ is common in women and in those with BN it induces high levels of anxiety [27,28]. However, no fMRI study has investigated whether or which alterations in brain function (e.g. in the insula), are associated with this phenomenological distinction between women with and without BN using self-schematic processing of other slim bodies.

In summary, to obtain information on the neurobiological underpinnings of binge-eating and body weight and shape concerns in BN, the neural correlates of processing of food and body image stimuli require further investigation. Accordingly, this fMRI study investigated brain activation patterns associated with processing of food stimuli and body image. We hypothesized that, compared to HCs, people with BN would 1) activate the medial prefrontal cortex and ACC more, and the DLPFC less in response to food stimuli; and 2) show more insular cortex and less medial prefrontal cortex activation when they compared their own appearance to that of slim women.

The current study differs from previous research in this area in two ways. First, we used a written instruction before each block of stimuli that aimed to engage participants to think about eating the food in the picture, or to stimulate self-schematic processing of their own body in comparison to pictures of thin women. Secondly, we used a baseline condition that consisted of looking at a cross (referred to here as low level baseline; LLB) instead of a reference condition (i.e. looking at another type of stimulus). Additionally, we recruited a large sample of patients before the start of treatment.

## Methods

### Participants

Twenty-one treatment-seeking adult right-handed women with a Diagnostic and Statistical Manual for Mental Disorders (DSM-IV-TR) diagnosis of BN were recruited from an Eating Disorders Outpatient Department (Maudsley Hospital, United Kingdom). The clinical diagnosis was confirmed by the use of Module H of the Structured Clinical Interview for DSM Axis I (SCID-I) Patient Version [29]. Exclusion criteria were: 1) The use of psychotropic medication; antidepressant treatment was allowed if the dose had not changed for at least 6 weeks prior to the scan; 2) A current or past neurological disorder; 3) The presence of a current comorbid Axis I disorder that was considered a primary diagnosis; decisions on this were based on agreement between two researchers (FVDE, US). Affective symptoms are common in people with BN, but their presence is not an exclusion criterion as long as they merely constituted a ‘mood or anxiety disorder *not otherwise specified’* (DSM-IV-TR) as part of the overall clinical presentation. Comorbidity was assessed with the SCID-I [30]; 4) Current suicidal behavior (clinical interview); 5) Pregnancy. Additional exclusion criteria based on MRI safety guidelines were applied to BN and HC participants.

Twenty-one adult right-handed female HCs were recruited through local advertisement in King’s College London. Exclusion criteria were: 1) The use of psychotropic medication; 2) Presence of a current or past history of an Axis I disorder [SCID-I Non-Patient Version] [29]; 3) Pregnancy; 4) Presence of significant eating disorder psychopathology [Eating Disorder Examination –Questionnaire score ≥2.80 [31]].

Ethical approval and written informed consent was obtained (The Joint South London and Maudsley and The Institute of Psychiatry NHS Research Ethics Committee 07/H0807/86).

### Clinical characteristics

Participants completed self-report questionnaires to assess levels of eating disorder pathology [EDE-Q [32]], trait and state food craving [Food Craving Questionnaire –Trait and State (FCQ-T/S) [33]], anxiety related to appearance [Social Appearance Anxiety Scale (SAAS) [34], Physical Appearance Comparison Scale (PACS) [35]] and affective symptoms and stress levels [Depression, Anxiety Stress Scale (DASS) [36]].

### Functional magnetic resonance imaging paradigms: ‘food’ and ‘body image’

Both paradigms used a block-design with three conditions: *food or body images*, *control* and a *LLB*. Images were presented on a rear-projection screen and viewed through a mirror system fitted to the head coil. Each paradigm included 15 (3 LLB, 6 control and 6 food/body) blocks. The LLB was a white cross in the middle of a black screen. In the *food* paradigm, pictures of highly palatable food (e.g. pizza, chocolate) were contrasted with non-edible (*control)* objects (e.g. stationery) and the LLB. The stimuli (not the LLB) were matched for color and complexity (based on ratings by five volunteers) and have been used in previous fMRI studies [10,12,13,37]. In the *body image* paradigm, slim [approximate body mass index (BMI) 18.5 kg/m^2^] bodies of other women (the head out of the shot), were contrasted with interior design pictures and the LLB. These stimuli have previously been used in fMRI studies in people with anorexia nervosa (AN) and HCs; the size of the images in the compression format JPG was used as an exploratory measure for objective visual complexity, and images of the body and interior design pictures were matched for visual complexity [17,38]. The images of slim female bodies and interior design were provided by a women’s magazine [17,38]. The images were selected from a larger database if they were rated by 38 healthy female volunteers as (1) easy to recognize, (2) interesting and (3) provoking anxiety in self-comparison [17,38]. The use of body images with the head out of shot is important as people with BN differentially process facial emotions [39]. Although a contrast of interest in this study was between the *food/body* and the LLB conditions (see Data analysis below), the *control* condition was included to allow post-hoc comparisons with previous findings [10-13].

Blocks lasted 36 seconds; in the food/body and control blocks, 12 pictures were presented back-to-back for 3 seconds each. The cross in the LLB condition was continuously presented for 36 seconds. All blocks were preceded by an 8-second (visual) instruction, specific for the type of stimulus: [LLB: “*watch the cross*”; food: e.g. “*imagine eating these foods*”; non-food: e.g. “*imagine using these tools*” [13]; body image: e.g. “*compare your own body against the bodies in the pictures*”; non-body image: e.g. “*compare the furniture against that in your own house*”]. Following all blocks, participants used a button box to complete two visual analogue scales (VAS) presented on the screen for 4 seconds each: “*how anxious do you feel*” and “*how much do you crave food now*”; scores ranged from 0 (“*not at all*”) to 10 (“*extremely*”) with the cursor starting point at 5. The total running time for each paradigm was 13 minutes.

### Procedures

Participants were asked not to eat or drink for two hours prior to the start of the study (water was allowed). The order of presentation was counterbalanced, i.e. *food* [BN: n = 10; HC: n = 10] or *body image* [BN: n = 11; HC: n = 13] first.

### Image acquisition

fMRI data were acquired on a 1.5T GE Signa MR system (GE Medical Systems, Milwaukee, Wisconsin). T_2_*-weighted gradient echo planar images depicting blood-oxygen-level-dependent (BOLD) contrasts were acquired every 2 seconds (repetition time) with an isotropic 3.3 mm × 3.3 mm in-plane resolution. The echo time was 40 ms, the flip angle was 80° and the matrix size was 64 × 64 voxels. Whole brain coverage was acquired with 29 slices (slice thickness 3mm, inter-slice gap 1 mm); 390 T_2_*-weighted whole brain volumes were acquired for each experiment. A whole-brain high resolution structural scan (inversion recovery gradient echo planar image), used for standard space normalisation, was also acquired in the inter-commissural plane with TE = 40 ms, TR = 3 s, flip angle = 90°, number of slices = 43, slice thickness = 3.0 mm, slice skip = 0.3 mm, in-plane voxel size = 1.875 mm, providing complete brain coverage. Data quality was assured using an automated quality control procedure [40].

### Data analysis

Between-group differences in demographic and clinical characteristics were examined using Chi^2^ and independent samples t-tests. To analyze VAS scores, repeated measures analyses of variance were conducted (VAS scores as within and group as between-subject factor). The reported effect size (ES) η_p_^2^ is calculated as [SS-between/(SS-between + SS-error)] (SS = squared sum) [0.01–0.06 = small; 0.06–0.14 = moderate; >0.14 = large [41]].

The imaging data were analyzed with the XBAM software (version 4.1) developed at King’s College London’s Institute of Psychiatry [42]. The non-parametric approach used in XBAM may be preferential in light of widespread departures from normality in fMRI group data [43]. After motion correction, the estimated BOLD effect was modeled by two Poisson functions with hemodynamic delays of 4 and 8 seconds. All participants were within acceptable limits for head movement (<1.0 mm). The least-squares model of the weighted sum of these two functions was compared with the signal in each voxel to obtain a goodness of fit statistic. The distribution of this statistic under the null hypothesis was calculated by multiple wavelet-based resampling of the time series and refitting the models to the resampled data. The contrasts reported here are between the food/body and LLB conditions.

Generic group activation maps (food/body vs LLB) were constructed by mapping the observed and randomized test statistics into standard space and computing and testing median activation maps. Median statistics were used to minimize the impact of outlier effects. Between-group differences were established by permuting data between groups to determine the sampling distribution of group differences under the null hypothesis. Group membership was permuted 1000 times, and a null distribution formed at each voxel, containing only the randomized voxel statistics at that voxel. The significance of each voxel was assessed against its own null distribution. An identical permutation strategy was applied at all voxels, meaning that it is valid to subsequently form clusters of spatially contiguous significant voxels.

3D-clusters were generated from the voxel-based statistical maps from the group and between-group analyses. The probability of occurrence of any cluster in the observed data was computed by reference to the computed null distribution [44]. Statistical thresholds within the analysis were adjusted so as to obtain less than one false positive 3D-cluster per map.

For both the *food* and the *body image* condition, within-group activation maps (by contrasting these conditions with LLB) for the BN and HC groups were first constructed. Then, between-group differences in activation in both conditions were investigated with a whole brain analysis approach. In addition, region-of-interest (ROI) analyses, using one-cm spherical ROIs centered on previously reported fMRI activation coordinates were carried out [10-13]. For the *body image* contrast, 3 ROIs of the same dimension were created centered on the coordinates for the left medial prefrontal cortex (BA9) and the left and right insula (BA13) reported by Mohr et al. (2011).

## Results

### Participant characteristics (Table 1)

**Table 1:** Participant demographic and clinical characteristics

	**HC (n = 23)**	**BN (n = 21)**	**HC vs BN**
Age (in yrs)	27.3 ± 5.1	28.0 ± 7.1	NS
BMI (kg/m2)	21.3 ± 2.4	23.4 ± 5.0	NS
Hours since last meal	3.6 ± 2.8	5.5 ± 4.3	NS
Number on OC	8/23 (35%)	6/21 (29%)	NS
Not on OC in follicular menstrual phase	9/15 (60%)	10/15 (67%)	NS
Number on medication, compound and dose	NA	9/21 (43%)	NA
		1 citalopram 20mg	
		1 citalopram 30 mg	
		3 fluoxetine 20 mg	
		1 fluoxetine 40 mg	
		2 fluoxetine 60 mg	
		1 venlafaxine 300 mg	
Number of smokers	2/23 (9%)	3/21 (14%)	NS
History of AN	NA	8/21 (38%)	NA
Duration of illness	NA	0–5 year (n = 3)	NA
		5–10 year (n = 12)	
		10–15 year (n = 3)	
		>15 year (n = 3)	
EDE-Q total	0.6 ± 0.5	4.8 ± 0.7	t_(1,43)_ = 24.4; p < 0.01
EDE-Q restraint	0.4 ± 0.5	4.5 ± 0.9	t _(1,43)_ = 18.7; p < 0.01
EDE-Q eating concerns	1.1 ± 0.3	4.2 ± 0.2	t _(1,43)_ = 17.0; p < 0.01
EDE-Q weight concerns	0.7 ± 0.7	5.1 ± 0.8	t_(1,43)_ = 19.8; p < 0.01
EDE-Q shape concerns	1.0 ± 0.8	5.3 ± 0.7	T_(1,43)_ = 19.1; p < 0.01
DASS-21 total (range 0–86)	19.0 ± 6.5	74.4 ± 29.0	t_(1,43)_ = 8.3; p < 0.01
DASS-depression (range 0–42)	5.4 ± 5.4	23.4 ± 11.6	t_(1,43)_ = 6.7; p < 0.01
DASS-anxiety (range 0–42)	3.4 ± 4.0	23.2 ± 11.4	t_(1,43)_ = 7.9; p < 0.01
DASS-stress (range 0–42)	11.2 ± 7.6	27.8 ± 9.6	t_(1,43)_ = 6.4; p < 0.01
FCQ-Trait (range 39–236)	77.6 ± 17.3	159.4 ± 27.1	t_(1,43)_ = 12.1; p < 0.01
FCQ-State (range 15–75)	25.4 ± 7.9	43.5 ± 12.3	t_(1,43)_ = 5.9; p < 0.01
SAAS (range 0–80)	26.7 ± 8.4	64.3 ± 11.0	t_(1,43)_ = 12.9; p < 0.01
PACS (range 0–25)	14.1 ± 3.6	21.3 ± 2.4	t_(1,43)_ = 7.7; p < 0.01

BMI: body mass index; EDE-Q: Eating Disorder Examination–Questionnaire; DASS: depression, anxiety and stress scale; FCQ: food craving questionnaire; OC: oral contraceptive; AN: anorexia nervosa; NS; not significant; NA: not applicable; SSRI: selective serotonin reuptake inhibitor; SAAS: social anxiety appearance scale; PACS: physical appearance comparison scale.

Participants’ demographic and clinical characteristic are reported in Table 1. Age, BMI, and the number of women on an oral contraceptive and smokers were similar in the BN and HC groups. The mean time from the last meal to the start of the scan was also comparable. Five in each group were assessed in the morning (10-12AM) and the others in the afternoon (4-6PM). Compared to HCs, women with BN had higher levels of eating disorder psychopathology (EDE-Q) and food craving (FCQ-S/T), higher stress and affective symptom levels (DASS-21), and more anxiety related to body appearance (SAAS, PACS). The mean number of binge episodes in the 28 days prior to enrolment was 26 (range: 8 to 85).

### Food paradigm (food vs low level baseline)

#### Whole-brain group activation maps (see Table 2 for details)

**Table 2:** ***Food *** **paradigm: group map activations in the bulimia nervosa (BN) and healthy control (HC) groups, and between-group differences in brain activation patterns for the contrast ‘food vs low level baseline (LLB)’**

	**p-value**	**Brodmann area**	**Talairach coordinates**	**Cluster size (voxels)**
**People with BN (n = 21)**				
***Food > LLB***				
L Superior Frontal Gyrus	0.004	BA 9	x = -3.6; y = 55.6; z = 29.7	46
L Medial Frontal Gyrus	0.003	BA 8	x = 0.0; y = 25.9; z = 36.3	59
L Lingual Gyrus	<0.001	BA 17	x = -14.4; y = -85.2; z = 0.0	1357
***LLB > Food***				
R Cerebellum, Culmen	0.002	-	x = 7.2; y = -37.4; z = -23.1	3657
**HCs (n = 23)**				
***Food > LLB***				
L Medial Frontal Gyrus	< 0.001	BA 6	x = -3.6; y = 11.1; z = 42.9	705
R Cuneus	< 0.001	BA 17	x = 21.7; y = -77.8; z = 9.9	1825
***LLB > Food***				
L Cingulate Gyrus	0.001	BA 24	x = -18.1; y = 3.7; z = 29.7	87
L Cerebellum	< 0.001	-	x = -7.2; y = -51.9; z = -23.1	6033
**People with BN (n = 21) vs HCs (n = 23) (food vs LLB contrast)**				
***People with BN > HCs***				
L Cuneus	< 0.001	BA17	x = 21.7; y = 77.7; z = 8.3	208
R Cuneus	< 0.001	BA17	x = -21.7; y = 77.7; z = 8.3	333
***HCs > People with BN***				
None				

The Talairach coordinates indicate the location of the peak voxel within each cluster.

The group activation maps in the BN and HC groups showed similar patterns i.e. increased activation in the middle frontal cortex and reduced activation in cerebellar regions when processing food stimuli compared to the LLB condition. In addition, people with BN engaged the superior frontal gyrus more when processing food compared to the LLB. The HCs engaged the ACC more in the LLB compared to the food condition (see Table 2 for details).

#### Whole brain analysis-group comparisons

The between-group comparison showed greater activation in the bilateral cuneus in people with BN compared to HCs (see Table 2 for details).

#### Region of interest analyses

No between-group differences were found.

#### Visual analogue scales (Figure 1)

**Figure 1:**
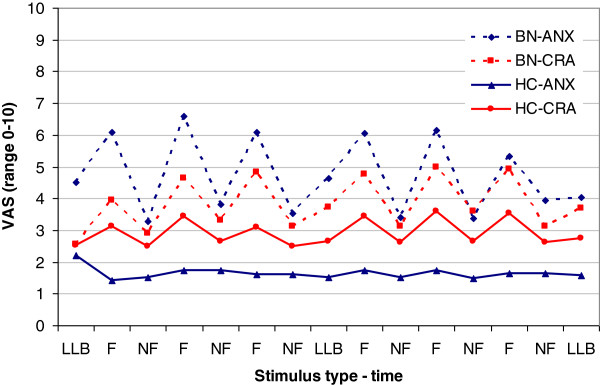
Visual analogue scale (VAS) scores for anxiety (ANX) and craving (CRA) following the low level baseline (LLB), non-food (NF) and food (F) condition in people with bulimia nervosa (BN) and healthy controls (HC).

Women with BN reported higher anxiety levels following exposure to visual food stimuli [F_(1,40)_ = 51.7; p < 0.001; ES = 0.56] and in the LLB condition [F_(1,40)_ = 23.2; p < 0.001; ES = 0.37], compared to HCs. In contrast, craving scores were not higher in the BN group in either the food [F_(1,40)_ = 3.3; p = 0.76; ES = 0.07] or LLB [F_(1,40)_ = 1.2; p = 0.28; ES = 0.03] condition.

### Body image paradigm (body image vs low level baseline)

#### Whole brain group activation maps (see Table 3 for details)

**Table 3:** ***Body image *** **paradigm: group map activations in the bulimia nervosa (BN) and healthy control (HC) groups, and between-group differences in brain activation patterns for the contrast ‘body image vs low level baseline (LLB)’**

	**Hemisphere**	**Brodmann area (BA)**	**Talairach coordinates**	**Cluster size (voxels)**
**People with BN**				
***Body image > LLB***				
R Middle frontal gyrus	< 0.001	BA9	x = 50.7; y = 7.4; z = 36.3	189
R Thalamus (pulvinar)	0.005		x = 25.3; y = -29.6; z = 3.3	42
L Inferior parietal lobe	0.004	BA40	x = -39.7; y = -40.7; z = 42.9	76
R Cerebellum, declive	< 0.001		x = 36.1; y = -55.6; z = -16.5	2259
***LLB > Body image***				
L Middle frontal gyrus	< 0.001	BA8	x = -28.9; y = 18.5; z = 42.9	689
R Frontal paracentral lobule	< 0.001	BA5	x = 18.1; y = -37; z = 49.5	410
R Cerebellum, culmen	< 0.001		x = 3.6; y = -40.7; z = -19.8	2706
**HCs**				
***Body image > LLB***				
R Middle frontal gyrus	< 0.001	BA9	x = 43.3; y = 11.1; z = 23.1	216
L Inferior frontal gyrus	0.005	BA6	x = -43.3; y = 0.0; z = 33.0	65
R Superior frontal gyrus	0.001	BA9	x = 3.6; y = 59.3; z = 33.0	158
R Inferior occipital	0.002	BA19	x = 39.7; y = -7.0; z = -6.6	2546
***LLB > Body image***				
L Cerebellum, culmen	0.002		x = -10.8; y = -44.4; z = -19.8	8199
**People with BN vs HCs**				
***People with BN > HCs***				
R Insula	0.002	BA13	x = 36.1; y = -18.5; z = -16.5	103
L Cerebellum, anterior lobe	0.003		x = -3.6; y = -55.6; z = -23.1	103
***HCs > People with BN***				
R Fusiform gyrus	< 0.001	BA37	x = 39.7; y = -66.7; z = -13.2	288
L Middle occipital cortex	< 0.001	BA19	x = 46.9; y = -63.0; z = -9.9	156
L Parietal cortex, precuneus	0.006	BA7	x = -21.7; y = -59.3; z = 39.6	47
R Parietal cortex, precuneus	0.006	BA7	x = 25.3; y = -55.6; z = 46.2	42

The Talairach coordinates indicate the location of the peak voxel within each cluster.

The group activation map from the BN group showed that when processing body image stimuli, compared to the LLB condition, parts of the frontal cortex were more (e.g. right middle frontal gyrus) and others less (e.g. left middle frontal gyrus) activated. Furthermore, in the BN group, body image processing resulted in increased activation in thalamic and parietal areas. In HCs, when processing body image stimuli, there was increased activation in various frontal areas (right middle and superior, and left superior frontal gyrus) and in the occipital cortex.

#### Whole brain analysis-group comparisons (see Table 3 for details)

**Figure 2:**
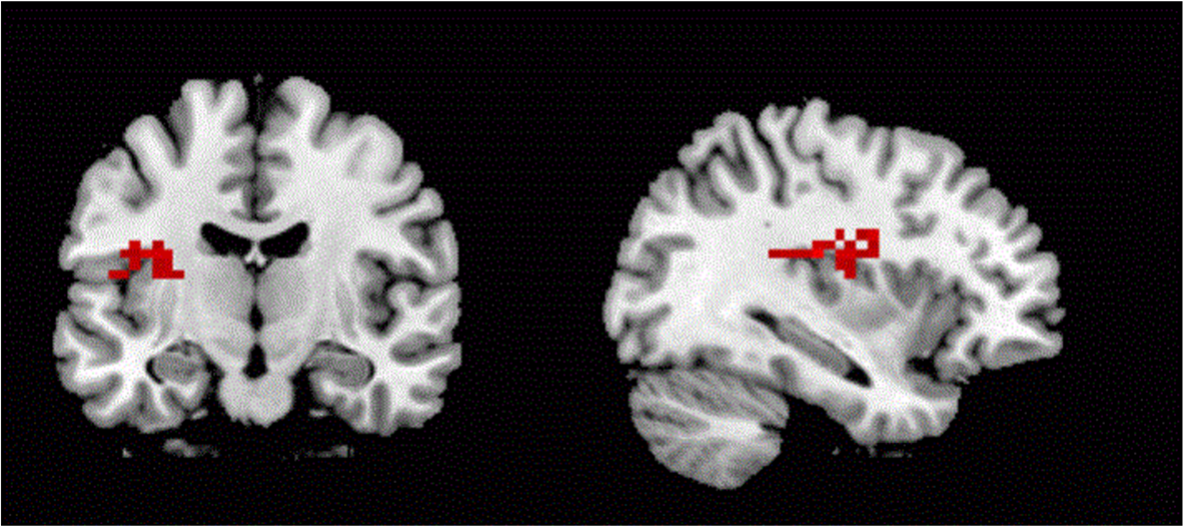
**Right insula increased activation in the right insula (BA13; x = 36.1; y = -18.5; z = -16.5) in people with bulimia nervosa when self-schematic processing of pictures of thin women, compared to healthy controls.** Contrast with low level baseline.

The between-group comparison showed greater activation in the right insula (Figure 2) and in the anterior lobe of the cerebellum in people with BN compared to HCs; less activation than the HC group was observed in the right fusiform gyrus, left middle occipital cortex and the (bilateral) precuneus.

#### Region of interest analyses

No between-group differences were found.

#### Visual analogue scales (Figure 3)

**Figure 3:**
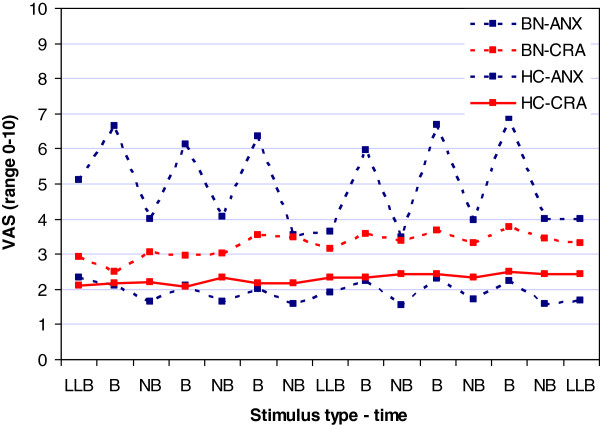
Visual analogue scale (VAS) scores for anxiety (ANX) and craving (CRA) following the low level baseline (LLB), non-body (NB) and body (B) condition in people with bulimia nervosa (BN) and healthy controls (HC).

Women with BN reported higher anxiety levels following exposure to body images [F_(1,40)_ = 63.1; p < 0.001; ES = 0.61] and in the LLB condition [F_(1,40)_ = 20.6; p < 0.001; ES = 0.81], compared to HCs. However, craving scores were not higher in the BN group in either the body image [F_(1,40)_ = 2.6; p = 0.12; ES = 0.06] or the LLB condition [F_(1,40)_ = 1.74; p = 0.19; ES = 0.04].

### Post-hoc analyses for the food vs control (non-food) and body vs control (non-body) comparisons (see Tables 4 and 5 for details)

**Table 4:** ***Food *** **paradigm: group map activations in the bulimia nervosa (BN) and healthy control (HC) groups, and between-group differences in brain activation patterns for the contrast ‘food vs non-food’**

	**p-value**	**Brodmann area (BA)**	**Talairach coordinates**	**Cluster size (voxels)**
**People with BN**				
***Food > Non-Food***				
R Lingual Gyrus	< 0.001	18	x = 14.4; y = -81.5; z = -9.9	710
R Thalamus (Pulivinar)	< 0.001	-	x = 10.8; y = -22.2; z = -6.6	68
R Anterior Cingulate	< 0.001	32	x = 3.6; y = 44.4; z = 6.6	203
***Non-Food > Food***				
R Cerebellum, Culmen	< 0.001	-	x = 10.8; y = -33.3; z = -13.2	191
L Precuneus	< 0.001	7	x = -3.6; y = -55.6; z = 29.7	40
R Middle Temporal Gyrus	0.002	21	x = 50.6; y = 0.0; z = -19.8	1578
L Middle Temporal Gyrus	< 0.001	37	x = -50.6; y = -55.6; z = 3.3	2063
**HCs**				
***Food > Non-Food***				
L Frontal Subcallosal Gyrus	0.002	34	x = 21.7; y = 3.7; z = -9.9	80
R Middle Frontal Gyrus	0.004	46	x = 43.3; y = 37.0; z = 19.8	54
R Lingual Gyrus	< 0.001	18	x = 14.4; y = -85.2; z = -3.3	1061
R Superior Parietal Lobule	0.002	7	x = 28.9; y = -55.6; z = 42.9	109
R Middle Frontal Gyrus	0.002	6	x = 43.3; y = 3.7; z = 46.2	72
L Frontal Superior Gyrus	0.001	8	x = -21.7; y = 25.9; z = 49.5	169
***Non-Food > Food***				
L Middle Frontal Gyrus	0.001	11	x = -43.3; y = 48.2; z = -13.2	45
L Caudate (Body)	0.002	-	x = -14.4; y = -14.8; z = 3.1	67
L Superior Temporal Gyrus	< 0.001	22	x = -36.1; y = -48.2; z = 9.9	3398
L Middle Frontal Gyrus	< 0.001	46	x = -39.7; y = 25.9; z = 16.5	513
**People with BN vs HCs**				
***People with BN > HCs***				
None				
***HCs > People with BN***				
None				

The Talairach coordinates indicate the location of the peak voxel within each cluster.

**Table 5:** ***Body image *** **paradigm: group map activations in the bulimia nervosa (BN) and healthy control (HC) groups, and between-group differences in brain activation patterns for the contrast ‘body vs non-body’**

	**p-value**	**Brodmann Area (BA)**	**Talairach coordinates**	**Cluster size (voxels)**
**People with BN**				
***Body image > Non-Body***				
Left Thalamus	< 0.001	-	x = -21.7; y = -29.6; z = 6.6	131
R Medial Frontal Gyrus	0.002	9	x = 25.3; y = 37.0; z = 9.9	56
L Anterior Cingulate	< 0.001	32	x = -18.1; y = 33.3; z = 13.2	163
R Inferior Frontal Gyrus	< 0.001	9	x = 46.9; y = 7.4; z = 26.4	217
L Precentral Gyrus	< 0.001	6	x = -50.6; y = 0.0; z = 29.7	78
R Inferior Temporal Gyrus	< 0.001	19	x = -46.9; y = -62.9; z = -3.3	1078
R Fusiform Gyrus	< 0.001	37	x = 43.3; y = -59.3; z = -6.6	1662
***Non-Body > Body image***				
L Insula	< 0.001	13	x = -32.5; y = 18.5; z = 6.6	301
R Superior Temporal Gyrus	0.002	22	x = 50.6; y = -48.2; z = 16.5	55
R Parahippocampal Gyrus	< 0.001	14	x = 25.3; y = -40.7; z = -9.9	4119
**HCs**				
***Body image > Non-Body***				
L Subcallosal Gyrus	0.003	25	x = -3.6; y = 3.7; z = -9.9	50
R Substantia Nigra	0.004	-	x = 18.1; y = -18.5; z = -6.6	49
R Thalamus, Dorsal Nucleus	0.006	-	x = 3.6; y = -18.5; z = 13.2	40
L Precentral Gyrus	0.005	9	x = -39.7; y = 3.7; z = 33.0	43
R Middle Temporal Gyrus	< 0.001	37	x = 43.3; y = -63.0; z = -3.3	1587
L Fusisform Gyris	< 0.001	37	x = -43.3; y = -63.0; z = -6.6	1011
R Inferior Frontal Gyrus	< 0.001	9	x = 43.3; y = 7.4; z = 29.7	318
***Non-Body > Body image***				
L Limbic Lobe, Uncus	< 0.001	28	x = -32.5; y = 3.7; z = -23.1	271
R Middle Temporal Gyrus	0.002	39	x = 39.7; y = -70.4; z = 23.1	88
L Parahippocampal Gyrus	< 0.001	36	x = -25.3; y = -37.0; z = -13.2	7408
***People with BN vs HCs***				
***People with BN > HCs***				
None				
***HCs > People with BN***				
L Fusiform Gyrus	0.002	37	x = -43.3; y = 63.0; z = -6.6	143
R Middle Temporal Gyrus	< 0.001	37	x = 43.3; y = 63.0; z = 3.3	245

The Talairach coordinates indicate the location of the peak voxel within each cluster.

These group comparisons show no brain activation differences between people with BN and HCs when they processed the food stimuli (Table 4). For the body vs non-body contrast, BNs had decreased activations in the left fusiform gyrus (BA37) and right middle temporal gyrus (BA37), compared to HC (Table 5).

### Baseline scanning-anxiety levels

Anxiety levels (VAS) at the start of the paradigms, prior to the presentation of the first food/body stimulus, were higher in the BN (4.9 ± 2.9) than the HC group (2.4 ± 1.9) (F_(1,42)_ = 6.3; p = 0.02).

## Discussion

This fMRI study investigated both food preoccupation/eating problems and body image concerns in the same clinical group. Specifically, in women with BN and in HCs, we investigated brain activation patterns associated with cognitive processing of both high caloric food images and pictures of other women’s thin bodies. Overall, our data suggest that the neural correlates of self-schematic processing of slim other women’s bodies differ between women with BN and HCs; in contrast, the neural correlates of processing visual food stimuli do not differ substantially.

With respect to the processing of food stimuli, difference between people with BN and HCs were limited. The bilateral cuneus was more activated in BN in response to the food stimuli. While little is known about the role of the cuneus in eating or other psychiatric disorders, there is indirect evidence that it may be involved in food associated reward, i.e. in a decision-making task with food cues, cuneus activity was related to both value and saliency [45]. Successful treatment may have an effect on the cuneus as recovered BN patients reportedly show less activation when given a taste of glucose [46]. Longitudinal studies with repeated assessments are required to establish whether treatment can have an effect on the role the cuneus plays in appreciation of food in people with BN.

The notion that dysfunctional fronto-striatal circuits underlie food and eating related psychopathology in people with BN, is not supported by our findings as we did not replicate previous findings of decreased DLPFC activity [10,12]. Two possible explanations for this relate to methodological aspects. First, the instructions on how to engage with food stimuli [those in the current study are the same as in other studies [10,12,15]] may impact on brain activation patterns. In the present study, instructions were adapted to the type of stimulus (food/body, control or LLB) and were repeated prior to each block to ensure continuous engagement throughout. In two previous studies, participants were instructed once, prior to the start of the experiment, with the following wordings “*You will be shown pictures of food and other objects. Look at each picture and think how hungry it makes you feel*” [10] and “*You will be shown pictures. Look at each picture attentively*” [12], respectively. It is conceivable that in the present study, the processing of stimuli was more consistent throughout the paradigm. Our instructions were the same as used by Brooks et al. (2011), but they were audio-recorded in that study. As in the present study, Brooks et al. (2011) did not report frontal or striatal activation differences between people with and without BN. While no study has directly investigated this ‘instruction’ effect in a within-subject comparison in people with an eating disorder, Siep et al. (2012) have demonstrated that brain activation in frontal and striatal regions can differ based on the instructions given, e.g. 1) to passively view foods, 2) to up-regulate food palatability thoughts, 3) to apply cognitive reappraisal (e.g., thinking about health consequences), or 4) suppress food palatability thoughts and cravings [47]. An indirect comparison with their findings suggests that the left medial prefrontal cortex activation seen in both our HC and BN group (Table 2) is in accord with the task to ‘up-regulate’ food palatability. However, our protocol most likely combines aspects of more than one of their instructions and this may explain why we do not find a difference in areas such as the DLPFC. Thus, the way the participants engaged with the visual stimuli in our and the Brooks et al. (2011) study may have been different from other studies that reported on altered frontal functioning [10,12].

A second explanation for the difference between our and previous results relates to the analysis. The current study includes a LLB to which the food condition is contrasted; others used food vs non-food contrasts. Our post-hoc food vs non-food contrast analyses yielded no clusters of brain activation differences between people with BN and HCs, despite activity alterations in several frontal areas within the individual (BN and HC) groups (Table 4). Thus, this does not replicate any of the previous findings and hence use of a LLB control condition does not explain differences between reports.

It is important to interpret the current data in the context of the subjective experiences which indicate that anxiety, and not craving levels, are higher in the BN group, compared to the HC group. These within-paradigm findings correspond with post-scanning assessment of the stimuli in previous studies [10,12,14]; craving was not assessed by Brooks et al. (2011). This is the first study in BN to assess craving and anxiety during the paradigm and it appears that food provokes more anxiety rather than more craving in people with BN (compared to HCs). It is possible that visual presentation of food stimuli is less salient than other forms of exposure such as real food or virtual reality [48]. For example, exposure to real food results in more craving in people with bulimic disorders than HCs [49]. Within-paradigm assessments of food craving in HCs showed increased craving only when people were instructed to think about the palatability of the food, but not in other conditions [47]; this supports the idea that the instruction that we used was more complex and may have resulted in suppression of urges to eat the food in both healthy and BN participants.

In contrast to the processing of food stimuli, an evaluative comparison of own body against slim women is associated with distinct brain activation patterns in women with and without BN. People with BN activated the insula more and the fusiform gyrus less; this indicates that–when comparing themselves to slim women–they focus more on their self/own body [i.e. heightened self-referencing [50]] and less on the actual ‘other’ body shape/contours. Our data are in accord with findings of increased insula activation in people with BN when rating satisfaction of ‘thin self-body images’ [20] and increased anterior insula activation for the desired low body size in patients with AN [17,51]. These data support hypotheses that propose that there is altered insula functioning during the integration of interoceptive information and emotion processing in people with eating disorders [26]. Less engagement of the fusiform gyrus, a key region in visual processing of bodies [52], in people with BN was also reported when line drawings of bodies were used [22]. Also, the left fusiform gyrus and the middle temporal gyrus may reflect a certain degree of body-related avoidance; interventions focused on body image do indeed show an increase in the Extrastriate Body Area (EBA) following successful treatment when patients look at other slim women [53]. We did not replicate the decreased medial prefrontal activation reported by others [18-21]; this is most likely due to the methodological differences related to the stimulus type [54] and to the instruction. Furthermore, as the medial prefrontal cortex is involved in self-referential processing and participants were requested to compare themselves to the women in the pictures, this may have led to an increased activity in the medial prefrontal cortex that counteracted the expected effect. Similar to the food paradigm, anxiety during the task was more prominent in the BN group and may have accentuated insula activation.

It is possible that anxiety related to the study procedures plays a role in participants’ behavior. People with BN reported higher anxiety levels following exposure to visual food and slim body stimuli, although craving levels did not differ. Participants with BN were more anxious at the start of the scanning session, but, it remains unclear whether this relates to the fMRI procedure [55,56] or to higher baseline stress and (anticipatory) anxiety.

To investigate the potential effects of antidepressant intake on the results, we analyzed brain activation patterns between participants on medication and those not on medication. The differences in the food (fusiform and lingual gyrus, and posterior cerebellum) and body image (lingual gyrus and anterior cerebellum) paradigm do not explain the main results of the current study. Detailed information is available from the corresponding author.

Strengths of this study are the use of two paradigms that relate to core BN psychopathology within the same group of participants and the assessment of subjective experiences in response to stimuli during the scanning. Secondly, the use of a LLB is beneficial; in its absence, the possibility that group differences already occur in the control rather than the active condition cannot be ruled out [17]. We note that Mohr et al. (2010) also used a fixation cross as baseline in their fMRI investigation of body image issues in people with BN and they also reported increased insula activation. Limitations include the lack of DSM Axis II assessment and, in the food paradigm, the use of various food types rather than an individualised set of stimuli. In addition, the current paradigm does not allow investigation of a contrast between high and low calorie foods: however, electrophysiological data show that people with BN have a high attentional bias towards food regardless of the caloric value [57]. For practical reasons, it was not possible to standardize the food intake prior to scanning. We did not study participants in the same menstrual phase but, an equal proportion in each group was on an oral contraceptive or in the follicular phase. It is also of note that the fMRI parameters may have resulted in only partial coverage of the cerebellum; hence these data should be interpreted with caution. ROIs in both paradigms were based on reported coordinates and were relatively small (in light of a conservative approach). Finally, this cross-sectional study is unable to differentiate between the ‘state’ or ‘trait’ nature of the findings.

## Conclusion

In summary, processing visual food stimuli and comparing oneself to other slim women elicits more anxiety, but not craving, in women with BN compared to HCs. Women with and without BN use similar brain structures to process food stimuli. When comparing their body against slim women, women with BN use the insula more (i.e. reflect more on themselves) and the fusiform gyrus less (i.e. look less at the other’s actual shape). This supports the idea that psychotherapy for BN should have a particular focus on body image and not solely focus on food and eating related issues. There is evidence that psychotherapy can alter brain activation patterns in response to visual body stimuli in people with eating disorders [53]. Another clinical implication of our findings is that they can guide the development of future directed interventions such as transcranial or deep brain stimulation (e.g. low frequency, i.e. inhibitory, transcranial magnetic stimulation to the insula may reduce body image concerns).

## Abbreviations

AN: Anorexia nervosa; BMI: Body mass index; BN: Bulimia nervosa; BOLD: Blood-oxygen-level-dependent; CBT-BN: Cognitive behavioral therapy for bulimia nervosa; EDE-Q: Eating disorder examination-questionnaire; fMRI: Functional magnetic resonance imaging; HC: Healthy control; LLB: Low-level baseline; SAAS: Social appearance anxiety scale; VAS: Visual analogue scale.

## Competing interests

The authors declare that they have no competing interest.

## Authors’ contributions

FVDE and VG have equally contributed to the manuscript; both were also involved in the design of the study and data analysis. FVDE also collected the data. US, RU, IC, AS and CA helped conceiving the study and participated in its design. POH has contributed to the writing of the manuscript. All authors read and approved the final manuscript.

## Authors’ information

FVDE was a research fellow in the Marie Curie Research Training Network INTACT (MRTN-CT-2006-035988) from 08/2007 to 07/2010.

RU consults for the World Health Organization. RU is supported by the European Commission Innovative Medicine Initiative Joint Undertaking (IMI-JU) grant number 115008.

US and AS receive salary support from the National Institute for Health Research (NIHR) [Mental Health Biomedical Research Centre at South London and Maudsley NHS Foundation Trust and King's College London]. The views expressed are those of the authors and not necessarily those of the NHS, the NIHR or the Department of Health.

## Pre-publication history

The pre-publication history for this paper can be accessed here:

http://www.biomedcentral.com/1471-244X/13/302/prepub
